# 

**Published:** 1894-10

**Authors:** 


### CONTENTS:

### SELECTIONS.

Article I.—The Matrix in Filling Teeth. By J. D. Patterson, D. D. S. - - - - - - - - - 241
" II.—Some Notes on the Etiology of Pyorrhœa Alveolaris. By Junis E. Cravens, D. D. S. - - - - - 244
" III.—Care of the Mouth in Sick Persons. - - - - 247
" IV.—Protrusion of the Upper Jaw. By R. Ottolengui, M. D. S. - - - - - - - - - 249
" V.—Tin. By Dr. J. R. Thompson. - - - - - 264
' VI.—Some Points on Bridgework. By Dr. H. J. Ray. - 270
" VII.—Dental Civilization. By A Traveled English Practitioner. - - - - - - - - - 272
" VIII.—The Southern Dental Association. - - - 276

### EDITORIAL, ETC.

Observe, Digest, Concentrate, Act. - - - - - - - 280

### MONTHLY SUMMARY.

The Value of Sugar and the Effect of Smoking on Muscular Work. 281

Why are We Weary. - - - - - - - 283

Extracting for Irregularity. - - - - - - 285

Dr. J. Austin Dunn's Syringe. - - - - - - 285

An Original Crown. - - - - - - - - 286

To Band a Logan Crown. - - - - - - - 286

Devitalizing Pulps. - - - - - - - - 287

To Obtund Sensitive Dentine. - - - - - - 287

Pulpitis. - - - - - - - - - 288

Blood Poisoning. - - - - - - - - 288

Mouth Poultice. - - - - - - - - 288

